# Wnt1 is epistatic to Id2 in inducing mammary hyperplasia, ductal side-branching, and tumors in the mouse

**DOI:** 10.1186/1471-2407-4-91

**Published:** 2004-12-15

**Authors:** Susan Marino, Claire Romelfanger, Yoshifumi Yokota, Roel Nusse

**Affiliations:** 1 Department of Developmental Biology, Howard Hughes Medical Institute, Beckman Center, Stanford University Medical School Stanford, CA 94305, USA; 2 Department of Biochemistry, Fukui Medical University, Shimoaizuki 23-3, Matsuoka, Fukui 910-1193, Japan

## Abstract

**Background:**

During pregnancy, the mammary glands from *Id2 *mutant animals are deficient in lobulo-alveolar development. This failure of development is believed to be due to a proliferation defect.

**Methods:**

We have asked whether functional *Id2 *expression is necessary for Wnt induced mammary hyperplasia, side branching, and cancer, by generating mice expressing a Wnt1 transgene in an *Id2 *mutant background.

**Results:**

We show in this work that forced expression of Wnt1 in the mammary gland is capable of overcoming the block to proliferation caused by the absence of *Id2*. We also show that Wnt1 expression is able to cause mammary tumors in an *Id2 *mutant background.

**Conclusions:**

We conclude that functional *Id2 *expression is not required for Wnt1 to induce mammary hyperplasia and mammary tumors.

## Background

Basic helix-loop-helix (bHLH) transcription factors such as MyoD, E12, and E47 are key regulators of gene expression and control many differentiation events during development [1-3]. These transcription factors bind E-box or E-box-like sequences as homo-or heterodimers, and control the transcription of target genes containing these sequences in their promoters. The HLH domains dimerize with each other, whereas the basic domains bind to DNA. The Id (Inhibitor of DNA binding) proteins are HLH proteins that lack a basic domain. Id proteins act as dominant inhibitors of bHLH transcription factors by blocking their ability to bind to DNA and activate gene transcription [2,3]. Since the bHLH proteins regulate cell-type specific gene expression during cell commitment and differentiation, the formation of inactive heterodimers of bHLH proteins with Id proteins inhibits the commitment and differentiation the bHLH proteins promote.

There are 4 mammalian Id genes, which show differences in their patterns of expression and function [2,3]. One of them, *Id2*, is expressed in glandular and ductal epithelium of the mouse mammary gland and has also been implicated in its development. Mammary glands of female mice that are homozygous mutant for *Id2 *have impaired lobulo-alveolar development [4]. In several tissues, including colon tumors induced by activation of the Wnt pathway, the expression of *Id2 *is regulated by Wnt-β catenin signaling [5,6]. It has been proposed that Wnt signaling may inhibit differentiation and promote the maintenance of a proliferative state by increasing *Id2 *expression, thereby leading to cancer. We have asked therefore whether functional *Id2 *expression is necessary for Wnt induced mammary hyperplasia, side branching and cancer, by generating mice expressing a Wnt1 transgene in an *Id2 *mutant background.

## Methods

We used heterozygous *Id2 *males and females on a 129/Sv background. *Id2 *genotyping was done by PCR (95C, 5 min; 62C, 1 min, 72C, 1 min, 95C,1 min, 30 cycles; 62C, 1 min, 72C, 5 min) using primers *Id2*-S (5'-tctgagcttatgtcgaatgatagc-3'), Id-2AS (5'-cgtgttctcctggtgaaatggctg-3'), and neo 1 (5'-tcgtgctttacggtatcgccgctc-3").

Hemizygous transgenic MMTV-Wnt1 males on a mixed FVB/BL6/SJL background were obtained from Yi Li in the H.Varmus laboratory. Genotyping was done by PCR (94C, 4 min; 94C, 45 sec, 55C, 30 sec, 72C, 60 sec, 30 cycles; 72C, 10 min) using Wnt1 (5'-gaacttgcttctcttctcatagcc-3') and SV40 (5'-ccacacaggcatagagtgtctgc-3') primers that produce a 350 bp product in transgenic mice.

### Carmine staining

Five mammary glands per mouse were removed and fat and muscle were dissected away. The glands were flattened between two slides and flooded with Carnoy's fixative (3:1 95% ethanol to glacial acetic acid) and fixed overnight. They were then de-fatted in 3 changes of acetone, rehydrated, stained overnight in 0.2% carmine and 0.5%KSO_4_, dehydrated, cleared in xylene, and mounted in Permount.

## Results

**Figure 1:**
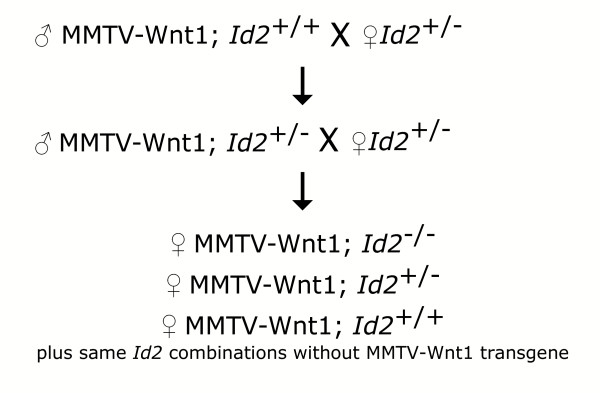
The Wnt1 transgene was crossed into an *Id2 *-/- background in order to produce cohorts of WT, *Id2 *+/-, and *Id2 *-/- females with and without the Wnt1 transgene. The scheme was designed so that mothers could feed their own young and so that all cohorts being compared would share a common background.

We used mice carrying a transgene in which Wnt1 is under the control of the promoter of the Mouse Mammary Tumor Virus (MMTV-Wnt1 Tg) [7] and we crossed these to *Id2 *loss of function mutant mice [4,8]. Crosses were set up to avoid reliance on *Id2 *-/- or Wnt1 transgenic mothers, as these animals cannot feed their young [4,7]. *Id2 *+/- females were crossed with MMTV-Wnt1 hemizygous transgenic males, producing 7 MMTV-Wnt1 Tg; *Id2*+/- males (Figure 1). These males were then crossed with *Id2 *+/- females to produce the experimental and control classes of virgin female mice: MMTV-Wnt1 Tg; *Id2 *-/-, MMTV-Wnt1 Tg ; *Id2 *+/-, and MMTV-Wnt1 Tg; *Id2*, as well as smaller numbers of animals in *Id2 *-/-; *Id2 *+/-; and WT classes. (Figure 1)

The subject animals were kept in mixed groups in autoclaved cages because *Id2 *-/- mice have an immunologic defect. Even with this care, 50% die before maturity [8]. *Id2 *-/- mice were born in sub-Mendelian ratios, they were smaller than litter-mates, and several died of unknown causes.

**Figure 2:**
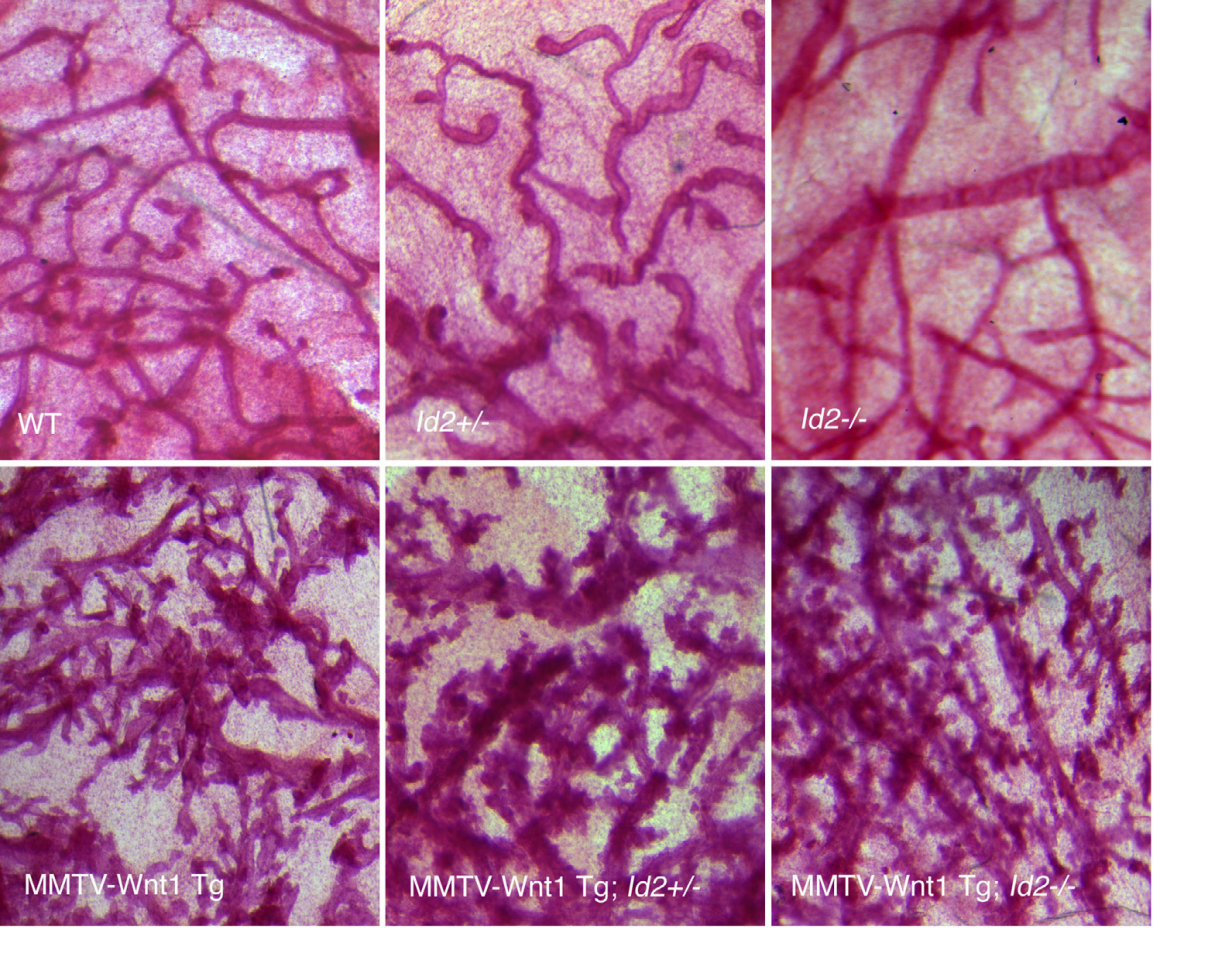
Carmine stained mammary gland whole mounts were made from virgin females. Top row, left to right: normal mammary gland development was observed in wild type (13 weeks), *Id2 *+/- (26 weeks), and *Id2*-/- (26 weeks) animals. Bottom row, left to right: Wnt1 induced hyperplasia and side branching were forced when the MMTV-Wnt1 transgene was present in wild type, *Id2*+/-, and *Id2 *-/- animals (all 19 weeks). Branching patterns are representative of a consistent phenotype observed in all mice and in both tumor-free glands and in glandular tissue associated with tumors in the MMTV-Wnt1 mice.

We examined the morphology of the mammary gland. At 3, 4.5, and 6 months the ductal branching patterns in normal mammary glands of 24 virgin mice from all six classes were examined in carmine stained whole mounts (Figure 2). As has been previously observed by others, the MMTV-Wnt1 Tg female glands had excessive ductal side branching compared to those of WT females [7] while *Id2 *+/- and *Id2 *-/- females glands were similar to those of WT females [4]. Glands from MMTV-Wnt1 Tg ; *Id2 *+/- and MMTV-Wnt1 Tg ; *Id2 *-/- mice had branching patterns resembling those of MMTV-Wnt1 Tg females at 3 months (Figure 2). At six months the three classes of transgenic mammary glands had very extensive, dense hyperplasia and side branching resembling that of pregnant wild type mice, while the six month virgin *Id2*+/- and *Id2*-/- glands had no additional branching and the six month virgin WT glands had moderate additional branching. Therefore, it appears that forced expression of Wnt1 in virgin mammary glands can overcome the absence of *Id2 *and lead to a highly branched ductal tree resembling the tree achieved normally during pregnancy.

**Figure 3:**
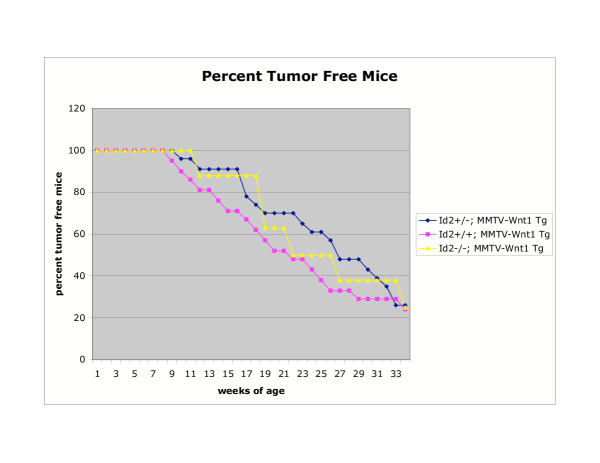
Three cohorts of mice, MMTV-Wnt1 Tg (n = 21), MMTV-Wnt1 Tg; *Id2 *+/- (n = 23), and MMTV-Wnt1 Tg; *Id2 *-/- (n = 8) were examined weekly by visual examination and palpation. No differences in tumor incidence were observed. MMTV-Wnt1 Tg : square; MMTV-Wnt1 Tg; *Id2 *+/- : diamond; MMTV-Wnt1 Tg; *Id2 *-/-: triangle.

In parallel, we examined tumor incidence in the animals. We excluded MMTV-Wnt1 Tg ; *Id2 *-/- females that died without developing tumors before 34 weeks, our endpoint, leaving only 8 animals in this group. Mice were examined and palpated weekly for mammary tumors. The smallest tumors detected using this method were 0.5 cm in diameter, but could be as large as 2 cm. The MMTV-Wnt1 Tg ; *Id2 *+/+ and MMTV-Wnt1 Tg ; *Id2 *+/- cohorts consisted of 21 and 23 females respectively. All three cohorts showed a similar rate of tumorigenesis (Figure 3). We concluded that Wnt1 is epistatic to *Id2 *in tumorigenesis, just as it is in promoting hyperplasia and side branching of the mammary gland (Figure 2).

## Discussion

The lack of *Id2*, a Wnt target, has severe consequences for mouse mammary gland development. During pregnancy, the *Id2 *mammary gland is deficient in lobulo-alveolar development. This failure of development is believed to be due to a proliferation failure rather than precocious differentiation of the mammary epithelia [4]. We show in this work that forced expression of Wnt1 in the mammary gland is capable of overcoming this block to proliferation. Although many targets of the Wnt pathway have been identified (see ), the mechanism through which hyperplasia and side branching is promoted by Wnt1 expression in the virgin mammary gland is unknown. Our results demonstrate that Wnt1 is not operating solely through *Id2 *or that it is not operating through *Id2 *at all.

Another known Wnt target with a similar loss of function phenotype is Cyclin D1 [9], a protein whose expression promotes advancement through the cell cycle and whose over expression results in hyperplasia and tumors in the mammary gland [10]. However, when Wnt1 is expressed in Cyclin D1 -/- mice, only a slight reduction in tumorigenesis is observed [11], suggesting that the Wnt1 pathway also does not operate primarily through Cyclin D1. Furthermore, over expression of Cyclin D1 did not promote lobulo-alveolar development in *Id2*-/- mice [12], a result that is in contrast to the dense side branching of the Wnt1 *Id2*-/- phenotype, suggesting that Wnt1 signaling is independent of both Cyclin D1 and *Id2 *in the mammary gland.

## Conclusions

By showing that forced expression of Wnt1 in the mammary gland is capable of overcoming the block to proliferation caused by the absence of *Id2*, we conclude that functional Id2 expression is not required for Wnt1 to induce mammary hyperplasia and mammary tumors.

## Competing interests

The author(s) declare that they have no competing interests.

## Author's contributions

SM and CR carried out the experiments. YY participated in the design of the study. SM and RN conceived of the study, participated in its design and coordination and wrote the manuscript. All authors read and approved the final manuscript.

## Pre-publication history

The pre-publication history for this paper can be accessed here:
